# Cold plasma technology as a novel antimicrobial platform: mechanisms, efficacy, and clinical translation: a systematic literature review

**DOI:** 10.1016/j.infpip.2026.100598

**Published:** 2026-09-22

**Authors:** Amir Namvar Vansofla, Mohammad Ebrahim Minaei, Neda Yousefi Nojookambari, Sajjad Yazdansetad

**Affiliations:** a Department of Biology, Faculty of Basic Sciences, Imam Hossein Comprehensive University, Tehran, Iran; b Department of Microbiology, School of Medicine, Shahid Beheshti University of Medical Sciences, Tehran, Iran

**Keywords:** Cold atmospheric plasma, Antimicrobial resistance, Reactive oxygen and nitrogen species, Biofilm eradication, Wound healing, Medical sterilization

## Abstract

**Background:**

The global antimicrobial resistance (AMR) crisis necessitates innovative therapeutic approaches beyond traditional antibiotics. Cold atmospheric plasma (CAP), an ionized gas containing reactive species, has emerged as a potent physical antimicrobial technology with broad-spectrum activity. This systematic review critically evaluates current evidence on CAP’s antimicrobial mechanisms, efficacy against diverse pathogens, and clinical translation potential.

**Methods:**

We conducted a systematic literature search following Preferred Reporting Items for Systematic Reviews and Meta-Analyses guidelines across PubMed, Scopus, and Web of Science databases (January 2018–December 2025). Included studies investigated CAP’s antimicrobial properties *in vitro*, *in vivo*, or clinically.

**Results:**

Analysis of 64 included studies revealed that CAP’s antimicrobial effects primarily stem from reactive oxygen and nitrogen species generation, causing oxidative damage to microbial membranes, proteins, and DNA. CAP demonstrates significant efficacy against multi-drug-resistant bacteria, viruses, fungi, and biofilms. Clinical studies show particular promise in chronic wound management, with randomized controlled trials demonstrating accelerated healing and reduced microbial load.

**Conclusion:**

CAP represents a promising multi-target approach to antimicrobial therapy, offering a multi-target mechanism that circumvents conventional resistance pathways. While standardization and dosimetry challenges remain, CAP holds immense potential for integration into clinical practice against AMR.

## Highlights

• Cold plasma’s multi-target mechanism circumvents antimicrobial resistance (AMR).
• Effective against multi-drug-resistant bacteria, biofilms, viruses, and fungi.
• Clinical trials confirm accelerated healing of chronic, infected wounds.
• Plasma-activated liquids enable novel therapeutic delivery strategies.
• A promising non-antibiotic approach to combat antimicrobial-resistance.

## Introduction

The relentless progression of antimicrobial resistance (AMR) represents one of the most serious threats to global public health, with recent estimates attributing nearly 5 million annual deaths directly to AMR [1]. The World Health Organization has repeatedly emphasized the critical need for novel antimicrobial strategies that operate through mechanisms distinct from conventional antibiotics [2]. Cold atmospheric plasma (CAP) technology has emerged as a particularly promising physical antimicrobial approach that meets this urgent need.

Plasma, often described as the fourth state of matter, consists of a partially ionized gas containing electrons, ions, photons, and various reactive species. The key innovation enabling biomedical applications is the development of ‘cold’ or non-thermal plasma systems that operate at atmospheric pressure and temperatures compatible with biological tissues (30–40°C) [3]. This thermal compatibility allows direct application to living organisms without causing thermal damage, while maintaining potent antimicrobial activity through the generation of reactive oxygen and nitrogen species (RONS).

The field of plasma medicine has evolved substantially since initial demonstrations of antimicrobial efficacy in the early 2000s. What began as surface decontamination technology has expanded into a multi-disciplinary field exploring applications in wound healing, cancer therapy, dentistry, and immunomodulation [4]. The unique advantage of CAP lies in its ability to deliver a simultaneous, multi-target assault on microbial pathogens, significantly reducing the likelihood of resistance development compared with single-target antibiotic therapies [5].

While several earlier reviews have addressed specific aspects of CAP – including hospital decontamination [6], disinfection technologies [7], bacterial load reduction [8], and biofilm interactions [9] – none have integrated recent mechanistic advances with a systematic comparison of efficacy across all major pathogen classes, updated clinical trials, biosafety, dosimetry, and economic considerations. Two recently published systematic reviews have further advanced the field by providing quantitative meta-analytic evidence for wound healing [10] and a comprehensive synthesis of dental applications [11]. The aim of the present review was to fill these gaps by providing a comprehensive, integrated, and up-to-date synthesis focused on AMR.

This systematic review provides a comprehensive and critical analysis of the current state of CAP technology as an antimicrobial platform, synthesizing the latest mechanistic insights, evaluating efficacy across diverse pathogen classes, and assessing the translational progress towards clinical implementation. Furthermore, we address key challenges in standardization and safety, while proposing future directions for research and clinical integration.

## Method

This systematic review was conducted in accordance with the Preferred Reporting Items for Systematic Reviews and Meta-Analyses (PRISMA) guidelines to ensure comprehensive and transparent reporting [12].

### Search strategy and information sources

We performed a systematic literature search across three major electronic databases: PubMed, Scopus, and Web of Science. The search period from January 2018 to December 2025 was selected to capture the most recent advances in the field as earlier comprehensive reviews have already summarized the foundational work on CAP antimicrobial activity. This review is a qualitative systematic review (systematic literature review) and does not include a meta-analysis because of substantial heterogeneity in plasma sources, treatment parameters, and outcome measures. We did not register a protocol with PROSPERO as the review focuses on a broad technology assessment rather than a single clinical outcome. The search used a combination of keywords and Medical Subject Headings terms, including: (‘cold plasma’ OR ‘non-thermal plasma’ OR ‘atmospheric pressure plasma’) AND (‘antimicrobial’ OR ‘antibacterial’ OR ‘antiviral’ OR ‘antifungal’) AND (‘reactive oxygen species’ OR ‘biofilm’ OR ‘wound healing’ OR ‘sterilization’).

### Eligibility criteria and study selection

Studies were included based on the following criteria: (1) original research articles investigating the antimicrobial properties of CAP; (2) in-vitro, in-vivo, or clinical studies; and (3) publication in English in peer-reviewed journals. Exclusion criteria included: reviews, conference abstracts, editorials, studies without full-text availability, and investigations using thermal plasma systems.

The study selection process involved initial screening of titles and abstracts, followed by full-text assessment of potentially relevant articles. Two independent reviewers conducted the selection process, with disagreements resolved through discussion or consultation with a third reviewer.

## Results

### Data extraction and quality assessment

An initial database search yielded 2750 records. After removal of duplicates and title/abstract screening, full-text review of the remaining articles led to the exclusion of 12 studies whose primary outcomes did not focus on antimicrobial efficacy (e.g. they examined only material properties or physical effects without microbial testing). The remaining 64 studies, all with confirmed antimicrobial efficacy outcomes (e.g. log reduction, clinical infection control, biofilm eradication), were selected for qualitative synthesis. The study selection process is summarized in the PRISMA flow diagram (Figure 1).

**Figure 1:**
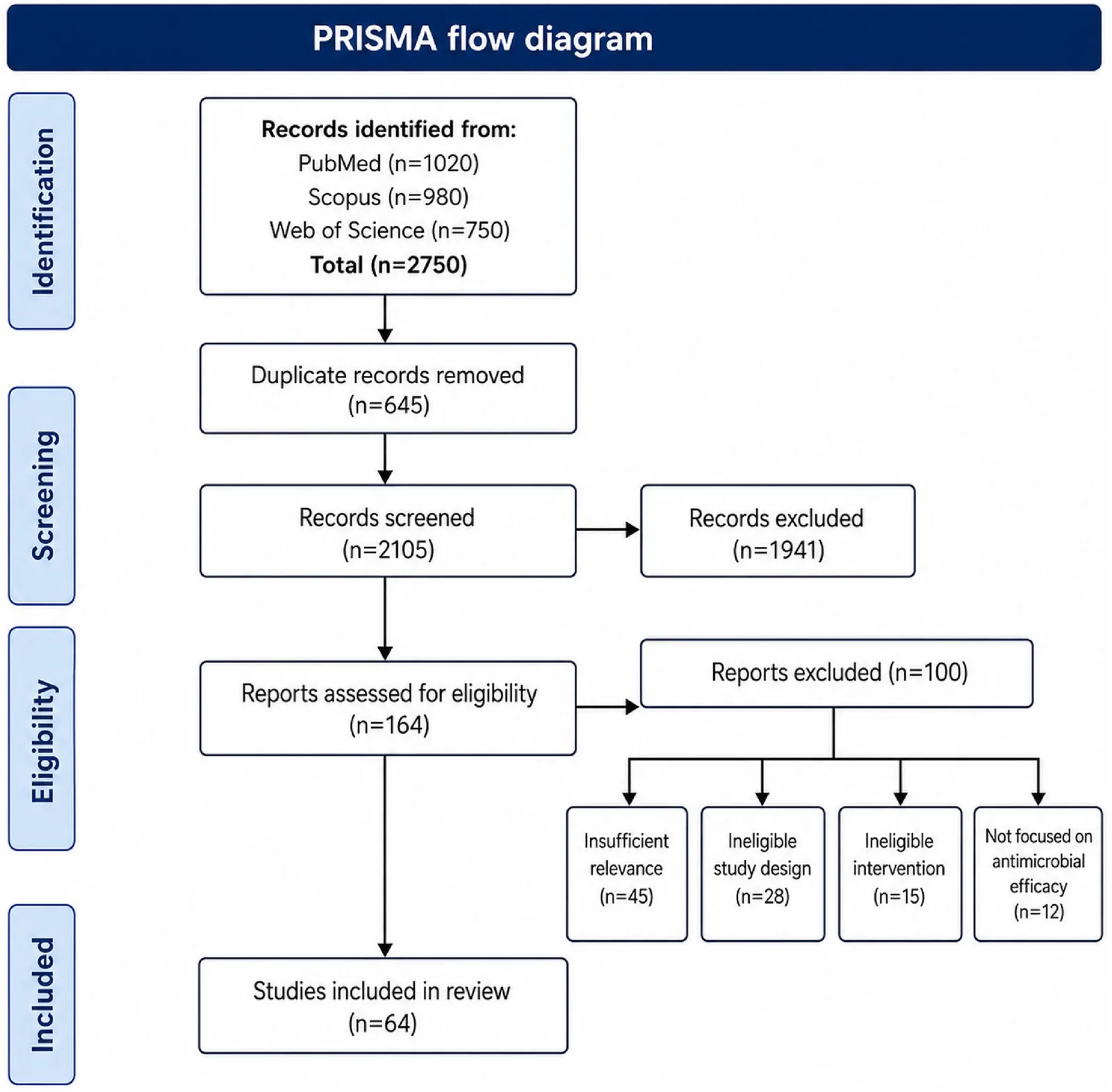
Preferred Reporting Items for Systematic Reviews and Meta-Analyses (PRISMA) flow diagram.

Data were extracted using a standardized form capturing information on study characteristics, plasma parameters, target micro-organisms, methodological approaches, key findings, and clinical outcomes. The quality of included studies was assessed, according to study design. For the few randomized controlled trials (RCTs) (*N* = 5) and animal studies (*N* = 12), we considered the principles of the Cochrane Risk-of-Bias tool and SYRCLE’s tool, respectively, but a full domain-by-domain table was not produced because the majority of included studies (*N* = 47) were in-vitro mechanistic studies for which these tools were not validated. Instead, we performed a qualitative assessment of study quality based on the clarity of methodology, reproducibility, appropriate controls, sample size, and consistency of findings.

### Mechanisms of antimicrobial action

The antimicrobial efficacy of CAP stems from the synergistic interaction of its physical and chemical components, with recent research providing deeper insights into the molecular mechanisms involved.

#### RONS generation and interactions

The primary antimicrobial activity of CAP is mediated by the complex mixture of RONS generated during plasma operation. Recent studies using advanced spectroscopic techniques have precisely characterized the temporal and spatial distribution of these species, revealing distinct RONS profiles depending on gas composition and power settings [13,14]. The RONS cocktail includes both short-lived radicals such as hydroxyl radicals (•OH), singlet oxygen (^1^O_2_), superoxide anions (O_2_·^−^), and nitric oxide (•NO), as well as long-lived species including hydrogen peroxide (H_2_O_2_), ozone (O_3_), nitrite (NO_2_^−^), and nitrate (NO_3_^−^) [15]. However, as noted by Karnopp *et al.* [15], none of the individual solutions replicated the comprehensive alterations observed in plasma-activated water, and even the composite solution containing all three RONS showed more pronounced changes but still did not fully match plasma-activated water’s properties. The relative concentration and biological effects of these species depend critically on the plasma source parameters and the environmental conditions.

At the molecular level, these reactive species induce comprehensive oxidative damage to essential microbial components. Membrane lipids undergo peroxidation through radical chain reactions initiated primarily by hydroxyl radicals, leading to loss of membrane integrity and increased permeability. Recent lipidomic studies on CAP-treated *Salmonella typhimurium* have identified specific oxidized phospholipid species that correlate with loss of membrane function, demonstrating the functional impact of CAP on bacterial membrane integrity [14]. Protein damage occurs through oxidation of cysteine thiol groups, methionine residues, and aromatic amino acids, resulting in protein unfolding, aggregation, and loss of enzymatic function. Proteomic analyses of CAP-treated bacteria reveal extensive carbonylation of key metabolic enzymes [13]. In addition, reactive species can penetrate the cell and cause oxidative damage to DNA, including base modifications, single-strand breaks, and formation of thymine dimers, leading to genotoxic stress and inhibition of replication. This multi-target oxidative assault, involving species such as hydroxyl radicals (•OH), ozone (O_3_), and nitric oxide (•NO), leads to membrane permeabilization, enzyme inactivation, and genotoxic stress, explaining the remarkably low potential for resistance development observed in serial passage experiments, where repeated sub-lethal CAP exposures over multiple generations failed to induce stable resistance [16].

#### Electric field effects and membrane interactions

Beyond chemical effects, the electric fields generated by CAP devices contribute significantly to antimicrobial activity through both direct and indirect mechanisms. The transient high-electric fields present in dielectric barrier discharge and plasma jet systems can induce electroporation, creating nanoscale pores in microbial membranes [17]. Recent research using real-time imaging and electrophysiological measurements has demonstrated that these pores facilitate the uncontrolled influx of extracellular RONS while allowing efflux of vital intracellular components, dramatically accelerating microbial inactivation [18]. The electroporation threshold varies with microbial size and membrane composition, with Gram-negative bacteria generally showing greater susceptibility than Gram-positive species due to differences in cell wall structure [19]. Furthermore, sub-electroporation electric fields can modulate membrane potential and affect vital membrane-associated processes including adenosine triphosphate synthesis and transport systems [20]. The combination of electric field effects with simultaneous RONS exposure creates a powerful synergistic antimicrobial approach that is difficult for micro-organisms to counteract through evolutionary adaptation.

#### Additional mechanisms and synergistic effects

Emerging research has identified several additional mechanisms contributing to CAP’s antimicrobial efficacy. The mild UV radiation emitted by certain plasma sources, though secondary to RONS effects, contributes to microbial inactivation through DNA damage, particularly in systems operating with specific gas mixtures [21]. More importantly, recent studies have revealed that CAP can trigger microbial apoptosis-like responses through activation of specific stress pathways and caspase-like proteases [22]. Additionally, CAP treatment has been shown to modulate microbial virulence by downregulating expression of key virulence factors and impairing quorum sensing systems in bacterial populations [23]. This antivirulence effect may contribute to the therapeutic outcome, even in situations where complete sterilization is not achieved. The comprehensive and multi-target nature of these antimicrobial mechanisms, encompassing cell wall damage, DNA damage, metabolic disruption, and biofilm eradication, provides a mechanistic basis for the broad-spectrum efficacy discussed in the following sections. Collectively, this indivisible synergy of physical and chemical insults presents a formidable barrier to the evolution of microbial resistance. Overcoming CAP’s multi-target action would require simultaneous adaptations in membrane composition, DNA repair, protein folding, and metabolic regulation, an evolutionary leap far less probable than the single-point mutations that confer antibiotic resistance.

Having elucidated the multi-target mechanisms that underpin CAP’s antimicrobial action, we now evaluate its empirical efficacy against a wide spectrum of planktonic and biofilm-embedded pathogens, including multi-drug-resistant strains.

### Efficacy against diverse pathogens

#### Antibacterial activity against planktonic and drug-resistant strains

CAP demonstrates potent and rapid bactericidal activity against both Gram-positive and Gram-negative bacteria, with efficacy extending to multi-drug-resistant strains that pose serious clinical challenges. Systematic analysis of in-vitro studies reveals consistent log reductions in viability exceeding a factor of 10^5^ within treatment times of 30 s to 5 min, depending on the specific plasma source and bacterial strain [24]. The clinical relevance of this log_10_reduction of factor 5 should be considered in the context of typical wound inocula. In-vitro studies often use high bacterial loads (10^6^–10^8^ colony-forming units [cfu]/mL or per surface) to challenge the technology under worst-case conditions. Chronic wounds, such as diabetic foot ulcers or venous leg ulcers, commonly harbour 10^5^–10^7^ cfu/g of tissue. Therefore, a log_10_reduction of factor 5 would lower the bacterial burden from, for example, 10^7^ cfu to <10^2^ cfu – a level generally considered below the threshold for clinical infection and compatible with wound healing. In clinical practice, CAP treatment times of 1–3 min per cm^2^ have been successfully used in RCTs, correlating well with a significant reduction of bioburden in infected diabetic foot ulcers [8,25]. Thus, the in-vitro log-reduction data align with clinically achievable outcomes. Against Gram-negative pathogens such as *Pseudomonas aeruginosa* and *Escherichia coli*, CAP induces rapid membrane damage due to the susceptibility of lipopolysaccharide components to oxidative modification [26]. Gram-positive organisms including *Staphylococcus aureus* show slightly different susceptibility patterns, with the thicker peptidoglycan layer providing initial protection that is overcome with longer exposure times [26].

Of particular clinical relevance is CAP’s undiminished efficacy against multi-drug-resistant organisms. Multiple studies have demonstrated potent activity against methicillin-resistant *Staphylococcus aureus*, vancomycin-resistant Enterococci, and carbapenem-resistant Enterobacterales [27,28].

The molecular basis for this lack of cross-resistance lies in CAP’s multi-target mechanism, which differs fundamentally from the specific molecular targets of conventional antibiotics [29]. Importantly, serial passage experiments designed to select for resistant mutants have consistently failed to generate strains with significantly reduced susceptibility to CAP, supporting the notion that resistance development is unlikely through conventional mechanisms. Most serial passage experiments have been short term (typically <20 generations) and performed under controlled laboratory conditions. Whether chronic, sub-lethal CAP exposure could select for resistant mutants over many generations in clinical settings remains unknown [16]. Therefore, the claim of ‘no resistance development’ should be viewed cautiously, and long-term evolutionary studies are needed.

#### Biofilm eradication and prevention

The ability of CAP to penetrate and disrupt microbial biofilms represents one of its most significant advantages over conventional antimicrobial approaches. Biofilms, characterized by structured communities of micro-organisms embedded in an extracellular polymeric substance, are notoriously resistant to antibiotics and contribute substantially to chronic and device-related infections [23]. CAP addresses this challenge through multiple synergistic mechanisms. Reactive species, particularly hydroxyl radicals, effectively degrade the polysaccharide, protein, and extracellular DNA components of the biofilm matrix [30,31]. This disruption of the extracellular polymeric substance compromises biofilm integrity and facilitates the penetration of reactive species to inactivate embedded cells.

Recent studies using confocal microscopy and microsensor technology have visualized this sequential process, showing initial matrix disruption followed by progressive inactivation of biofilm-associated cells from the surface inwards [31]. The efficacy against biofilms extends to mixed-species communities and fungal biofilms, with demonstrated activity against *micro-organisms* [32]. Comparative studies have shown that CAP can achieve biofilm eradication where conventional antiseptics like chlorhexidine fail, particularly in models mimicking the in-vivo environment [33]. Furthermore, sub-lethal CAP treatment has been shown to inhibit biofilm formation by interfering with initial attachment and quorum sensing mechanisms [34]. This antibiofilm activity has profound implications for managing chronic wounds, medical device–related infections, and dental caries, where biofilms play a central role in pathogenesis and treatment failure.

The broad-spectrum and potent antimicrobial capability of CAP, as evidenced by its efficacy against resilient biofilms, extends systematically across all major classes of pathogens. To provide a consolidated and comparative overview of this extensive activity, Table I summarizes the key parameters and outcomes documented in the literature. This systematic synthesis delineates the efficacy of CAP against Gram-positive and Gram-negative bacteria (including multi-drug-resistant strains), viruses, fungal pathogens, and bacterial spores, detailing typical treatment conditions, the achieved log-reduction values, and the primary physicochemical mechanisms responsible for microbial inactivation. Furthermore, the table critically highlights the distinct advantages of CAP over conventional antimicrobial therapies, such as its ability to circumvent established resistance mechanisms, its use in sterilizing heat-sensitive materials, and its capacity for rapid surface and aerosol decontamination. CAP devices can be broadly classified into two common types: dielectric barrier discharge (DBD) – where plasma is generated between two electrodes separated by an insulating dielectric layer – and atmospheric pressure plasma jet (APPJ) – where gas is flowed through an electric discharge region to produce a plasma plume that can be directed on to a target. These configurations influence the reactive species mix and treatment area, as summarized in Table I.

**Table I:** Comparative efficacy of cold atmospheric plasma (CAP) against different pathogen types

Pathogen class	Exemplary pathogens	Typical CAP treatment parameters	Efficacy (Log_10_ reduction)	Primary mechanisms of action	Key advantages over conventional approaches	References
Gram-negative bacteria	*Pseudomonas aeruginosa*, *Escherichia coli*, *Klebsiella pneumoniae*	APPJ, 1–3 min, 2–5 mm distance, He/air	4–6 log_10_	• Lipid peroxidation of LPS-containing outer membrane. • Protein carbonylation and enzyme inactivation. • Electroporation-enhanced RONS influx.	• Rapid action against multi-drug-resistant (MDR) strains. • Effective on surfaces and in liquid suspensions. • No acquired resistance reported in serial passage studies.	[35,36]
Gram-positive bacteria	*Staphylococcus aureus* (MRSA), *Enterococcus faecium* (VRE)	DBD, 2–5 min, 1–10 W, air	3–5 log_10_	• Oxidative degradation of peptidoglycan layer. • Membrane lipid peroxidation. • DNA strand breaks and oxidative base damage.	• Overcomes thick peptidoglycan barrier that limits some antibiotics.• Retains efficacy against MRSA and VRE. • Synergistic effect when combined with certain antibiotics.	[37]
Bacterial biofilms	*S. aureus*	APPJ/DBD, 5–10 min, direct exposure	3–4 log_10_ (in biomass and viability)	• Oxidative degradation of extracellular polymeric substance (EPS). • Disruption of quorum sensing signals. • Progressive inactivation of embedded cells postmatrix disruption.	• Eradicates biofilms where antibiotics fail due to poor penetration. • Physical disruption of matrix complements chemical killing. • Prevents biofilm recurrence more effectively than biocides alone.	[38]
Enveloped viruses	SARS-CoV-2	DBD, 30 s–2 min, ambient air	≥4 log_10_ (pfu/TCID_50_)	• Peroxidation of lipid envelope, leading to disintegration. • Oxidation of surface glycoproteins (e.g. spike protein). • Oxidative damage to viral genomic RNA/DNA.	• Broad-spectrum activity independent of viral strain mutations. • Rapid surface and aerosol decontamination capability. • Non-corrosive to materials, unlike some chemical disinfectants.	[39,40]
Non-enveloped viruses	Adenovirus, norovirus	APPJ, 2–5 min, He/O_2_ mixture	3–5 log_10_ (pfu/TCID_50_)	• Oxidation of capsid proteins, impairing host cell recognition/binding. • Direct damage to viral nucleic acid.	• Effective against highly stable viruses resistant to desiccation and many disinfectants. • Mechanism of action is physical–chemical, not reliant on viral replication cycle.	[41,42]
Fungal pathogens	*Candida albicans,* dermatophytes	APPJ, 3–8 min, He/O_2_	3–5 log_10_	• Oxidation of ergosterol in the fungal membrane. • Oxidative damage to cell wall components (chitin, glucans). • Triggering of apoptosis-like programmed cell death.	• Overcomes resistance to azole and echinocandin antifungals. • Effective against both planktonic cells and fungal biofilms. • Topical application allows targeted treatment with minimal systemic side-effects.	[43]
Bacterial spores	*Bacillus atrophaeus, Clostridium difficile*	DBD, 5–15 min, High Power, Air	2–4 log_10_	• Etching and gradual erosion of the protective spore coats. • Oxidative damage to inner membrane and core enzymes. • Degradation of dipicolinic acid (DPA) in the core.	• Sporicidal activity at low temperatures, unlike autoclaving. • Suitable for sterilizing heat-sensitive medical devices and materials. • Does not leave toxic chemical residues like ethylene oxide.	[44,45]

CAP, cold atmospheric plasma; APPJ, atmospheric pressure plasma jet; He, helium; LPS, lipopolysaccharide; RONS, reactive oxygen and nitrogen species; MDR, multi-drug-resistant; MRSA, methicillin-resistant *Staphylococcus aureus*; VRE, vancomycin-resistant *Enterococcus*; DBD, dielectric barrier discharge; EPS, extracellular polymeric substance; SARS-CoV-2, severe acute respiratory syndrome coronavirus 2; pfus, plaque-forming units; TCID_50_: 50% tissue culture infective dose; DPA, dipicolinic acid.

#### Antiviral applications and mechanisms

The antiviral properties of CAP have gained increased attention, particularly in the context of emerging viral pathogens and pandemic preparedness. CAP demonstrates efficacy against both enveloped and non-enveloped viruses through distinct but complementary mechanisms. For enveloped viruses such as severe acute respiratory syndrome coronavirus 2 (SARS-CoV-2), influenza virus, and herpes simplex virus, the lipid bilayer envelope is highly vulnerable to RONS-mediated lipid peroxidation [[46], [47], [48]]. Recent studies have shown that CAP treatment rapidly disrupts viral envelopes, leading to loss of structural integrity and infectivity [49]. This mechanism has proven effective against SARS-CoV-2, with multiple studies demonstrating rapid inactivation on surfaces and in aerosols using various plasma sources [50,51].

For non-enveloped viruses including adenovirus and norovirus, the protein capsid represents the primary target. RONS induces oxidation of critical amino acid residues in capsid proteins, leading to conformational changes that impair host cell recognition and entry [39]. Additionally, both enveloped and non-enveloped viruses suffer oxidative damage to their genomic material (RNA or DNA), preventing replication even if initial attachment occurs [39]. The broad-spectrum nature of this mechanism suggests potential use against emerging viral threats where specific therapeutics may not be immediately available. Recent applications have extended to antiviral surface coatings using plasma polymerization and the development of plasma-based air purification systems for continuous disinfection of healthcare environments [52].

#### Antifungal and sporicidal efficacy

CAP demonstrates significant antifungal activity against clinically relevant fungal pathogens including *Candida* species, dermatophytes, and emerging resistant fungi. The complex fungal cell wall, containing chitin and β-glucans, presents a more substantial barrier than bacterial membranes, necessitating longer treatment times for complete inactivation [[53], [54], [55]]. However, oxidative damage to membrane lipids (particularly ergosterol in fungal membranes) and cell wall components ultimately compromises cellular integrity [56]. Clinical studies have shown promising results for CAP in treating superficial fungal infections including onychomycosis and cutaneous candidiasis, with outcomes comparable to those of conventional antifungal therapies but with fewer side-effects and no risk of developing drug resistance [[57], [58], [59]].

Of particular note is CAP’s sporicidal activity against bacterial endospores from *Bacillus* and *Clostridium* species. Bacterial spores are among the most resistant biological structures known, capable of surviving extreme conditions that readily kill vegetative cells [60,61]. CAP overcomes this resistance through gradual erosion of the spore’s protective coats by reactive species, followed by oxidative damage to the inner membrane and core components [61,62]. This sporicidal activity has important implications for sterilization applications in medical and food industry settings, where elimination of spores is often the benchmark for effective sterilization [63].

### Clinical applications and translational progress

#### Wound management and infection control

The most advanced clinical application of CAP is in the management of chronic and infected wounds. Multiple RCTs have demonstrated that adjunctive CAP treatment significantly improves healing outcomes in diabetic foot ulcers, venous leg ulcers, and pressure ulcers [8]. The therapeutic benefit stems from the dual action of microbial reduction and direct stimulation of healing processes. CAP treatment consistently reduces bacterial load in infected wounds, including those colonized by multi-drug-resistant organisms [64]. Simultaneously, CAP modulates the wound microenvironment by enhancing epithelialization, promoting angiogenesis, and regulating inflammation [65]. Recent mechanistic studies have elucidated the molecular basis for these pro-healing effects. CAP treatment has been shown to activate the nuclear factor erythroid 2–related factor 2 (Nrf2) antioxidant pathway in human keratinocytes and fibroblasts, enhancing cellular resilience to oxidative stress while promoting proliferation and migration [66]. Additionally, CAP modulates cytokine expression patterns in wound beds, reducing pro-inflammatory signals while maintaining necessary immune responses [65,67]. The combination of antimicrobial and healing-stimulatory effects positions CAP as a comprehensive wound therapy rather than simply a disinfectant. Commercial medical plasma devices have received regulatory approval in multiple regions specifically for wound care applications, representing a significant milestone in the clinical translation of this technology [68].

Emerging strategies combining CAP surface modification with piezoelectric biomaterials have shown synergistic effects: CAP treatment of piezoelectric poly-L-lactide films, combined with zinc oxide (ZnO) filler, enables ultrasound-activated antimicrobial activity while simultaneously promoting keratinocyte proliferation and migration – effects that are directly relevant to skin regeneration and wound healing [69].

The quantitative evidence for CAP in wound healing has recently been consolidated in a meta-analysis of 12 RCTs, which reported significant reductions in wound area (mean difference [MD] = −1.36; 95% confidence interval [CI]: −2.09 to −0.62; *P* = 0.0011) and bacterial load (MD = −0.36; 95% CI: −0.64 to −0.08; *P* = 0.0126), with a healing rate ratio of 2.90 (95% CI: 1.45–5.80; *P* = 0.0052) [10].

#### Dental and oral medicine applications

CAP technology is revolutionizing multiple aspects of dental practice through its ability to access and decontaminate complex anatomical sites while preserving underlying tissues. In endodontics, CAP and plasma-activated irrigation solutions effectively eliminate *Enterococcus faecalis* biofilms from root canal systems, addressing a major cause of endodontic treatment failure [70]. In periodontics, CAP application reduces periodontopathogens and improves clinical parameters including probing depth and bleeding on probing [71]. The application extends to implant dentistry, where CAP treatment of titanium implant surfaces simultaneously decontaminates the surface and enhances osseointegration by improving osteoblast response [72]. Recent innovations include the development of specialized plasma devices for intra-oral application and the use of plasma-activated water as an antimicrobial rinse [73]. The latter approach offers particular advantages for patient self-care and management of oral conditions between professional treatments. Studies have demonstrated efficacy against cariogenic biofilms, oral candidiasis, and viruses associated with oral lesions [74]. The non-thermal nature of CAP allows treatment of sensitive oral tissues without causing pain or thermal damage, enhancing patient acceptance and compliance [73]. A systematic review of 19 studies (11 in-vitro, 7 animal, and 1 human RCT) confirmed that CAP demonstrates consistent antimicrobial activity against oral pathogens, enhances bonding and remineralization, and shows no adverse histological effects; however, clinical evidence remains limited, and routine use cannot yet be recommended [11]. The promising in-vitro efficacy of CAP has been progressively translated into tangible clinical applications across a diverse range of medical specialties, supported by a growing body of human trials. To provide a clear and systematic summary of this translational progress, Table II consolidates the major clinical applications of CAP, categorizing them by medical field and critically assessing the strength of the available evidence. The table delineates the specific clinical indications for which CAP has been used, from the management of chronic wounds and periodontitis to the palliative care of oncological lesions and the treatment of dermatological conditions like atopic dermatitis. For each application, it details the typical treatment parameters, including device type, duration, and frequency, employed in clinical reports, thereby offering practical guidance for implementation. Furthermore, the table synthesizes the key reported outcomes, such as significant reductions in bacterial load, accelerated wound healing, and improved patient symptoms, while directly linking these findings to validating references from major scientific databases. By including the level of clinical evidence, ranging from level Ia RCTs for chronic wounds to emerging level IV/V reports in ophthalmology, Table II provides an at-a-glance assessment of the current state of clinical validation for each application, highlighting both established successes and promising future directions for CAP technology in medicine.

**Table II:** Clinical applications of cold atmospheric plasma (CAP) with evidence levels

Clinical area	Level of evidence	Specific indication	Typical treatment parameters	Key reported outcomes	References
Wound care	Level Ia (RCTs)	• Chronic wounds (diabetic foot ulcers, venous leg ulcers)	• Device: kINPen® MED, PlasmaDerm® • Duration: 1–3 min/cm^2^ • Frequency: 2–3 times/week for 4–8 weeks • Gas: argon/air	• Significant reduction in bacterial load (including MRSA). • Accelerated wound closure and epithelialization. • Reduction in wound area by >50% vs control groups. • Improved granulation tissue formation.	[75]
Dermatology	Level II (cohort studies)	• Atopic dermatitis • Onychomycosis (fungal nail infection)	• Device: DBD, plasma jet • Duration: 1–2 min/site for AD; 3–5 min/nail for onychomycosis • Frequency: Daily to twice weekly for several weeks	• Reduction in SCORAD index, itching, and disease severity for AD. • Mycological cure and clinical improvement in onychomycosis comparable to those in topical antifungals. • Excellent tolerability and safety profile.	[76]
Dentistry	Level Ib (RCTs) and II (cohort studies)	• Periodontitis • Root canal disinfection • Peri-implantitis	• Device: various APPJs, DBDs • Duration: 30 s–2 min/site • Frequency: single session or repeated. • Gas: helium, argon/air	• Significant reduction in periodontal pathogens (Porphyromonas gingivalis, Aggregatibacter actinomycetemcomitans). • Improved clinical attachment level and reduced probing depth. • Effective eradication of Enterococcus faecalis biofilms in root canals. • Decontamination of implant surfaces.	[77]
Oncology (Palliative and adjuvant)	Level II/III (cohort studies/case-control)	• Advanced head and neck cancers • Palliative wound care in fungating tumours	• Device: kINPen® MED • Duration: 1–2 min/cm^2^ • Frequency: 2–3 times/week • Gas: argon	• Significant reduction in malodour and exudate from fungating lesions. • Improved local infection control. • Patient-reported reduction in pain. • Induction of apoptosis in superficial cancer cells.	[78]
Surgery and infection control	Level II/IV (cohort studies/case series)	• Pre-operative skin antisepsis • Decontamination of heat-sensitive surgical instruments	• Device: DBD, plasma jets • Duration: 30 s–2 min • Gas: air, helium	• Significant reduction of skin flora comparable to that of standard alcoholic antiseptics. • Effective inactivation of biofilms on endoscopic and polymeric medical devices. • No damage to sensitive materials.	[76]
Ophthalmology (Emerging)	Level IV/V (case reports/preclinical)	• Bacterial keratitis • Fungal keratitis	• Device: Microplasma jets • Duration: 30–60 s • Parameters: Very low power, precise application	• Successful treatment of antibiotic-resistant bacterial and fungal keratitis in case reports. • Reduction of microbial load without significant damage to corneal epithelium in preclinical models.	[79]

Evidence levels: Ia: evidence from meta-analysis of RCTs; Ib: evidence from at least one RCT; II: evidence from at least one well-designed controlled study without randomization; III: evidence from well-designed non-experimental descriptive studies (comparative, correlation); IV: evidence from expert committee reports or opinions and/or clinical experience of respected authorities; and V: evidence from preclinical or in-vitro studies.CAP, cold atmospheric plasma; RCT, randomized controlled trial; MRSA, methicillin-resistant *Staphylococcus aureus*; DBD, dielectric barrier discharge; SCORAD, SCORing Atopic Dermatitis; AD, atopic dermatitis; APPJ, atmospheric pressure plasma jet.

#### Medical device sterilization and surface decontamination

The non-thermal nature of CAP makes it ideally suited for sterilization of heat-sensitive medical devices and equipment. CAP effectively inactivates pathogens including bacterial spores on surfaces of polymers, metals, and ceramics without compromising material integrity or leaving toxic residues [63]. This has particular relevance for complex medical instruments such as endoscopes that cannot withstand conventional heat sterilization methods [80]. Recent technological advances have focused on developing plasma-based sterilization systems for clinical settings, with some systems achieving regulatory clearance for specific medical device applications [81]. Beyond instrument sterilization, CAP technology shows promise for continuous surface decontamination in healthcare environments. Plasma-based air purification systems can reduce airborne microbial loads, potentially decreasing transmission of respiratory pathogens in clinical settings [82]. Similarly, plasma-treated surfaces and coatings can provide persistent antimicrobial activity on high-touch surfaces in hospitals [21]. The ongoing coronavirus disease 2019 (COVID-19) pandemic has accelerated interest in these applications, with several studies demonstrating efficacy against coronaviruses on various surfaces [51]. The integration of CAP systems into hospital infrastructure represents a promising approach to reducing healthcare-associated infections (HAIs) through environmental control.

Recent advances have extended this concept to the direct deposition of antibacterial nanocomposite coatings on to textile and polymeric surfaces using CAP-based in-situ polymerization, enabling the fabrication of durable, biocompatible antimicrobial surfaces for wearable and implantable medical devices [83]. Similarly, CAP-assisted deposition of ammonium-functionalized glass ceramic coatings has been demonstrated on a wide range of polymeric and metallic surfaces, providing broad-spectrum antibacterial efficacy against common implant-associated pathogens while maintaining biocompatibility [84].

#### Plasma-activated liquids for therapeutic applications

A significant innovation in plasma medicine is the development of plasma-activated liquids as a means of delivering the therapeutic effects of plasma to sites not accessible to direct treatment. When liquids such as water, saline, or cell culture media are exposed to CAP, they acquire stable concentrations of long-lived reactive species, including hydrogen peroxide, nitrite, and nitrate [85]. These plasma-activated liquids retain antimicrobial activity for extended periods and can be applied topically, used for irrigation, or in some cases administered internally [85].

The therapeutic applications of plasma-activated liquids are rapidly expanding. A significant innovation in plasma medicine is the development of plasma-activated liquids as a means of delivering the therapeutic effects of plasma to sites not accessible to direct treatment. When liquids such as water, saline, or cell culture media are exposed to CAP, they acquire stable concentrations of long-lived reactive species, including hydrogen peroxide, nitrite, and nitrate. These plasma-activated liquids retain antimicrobial activity for extended periods and can be applied topically, used for irrigation, or in some cases administered internally [85].

The antimicrobial applications of plasma-activated liquids are rapidly expanding. In dermatology, plasma-activated solutions are being investigated for treatment of skin infections and inflammatory conditions [86,87]. The ability to standardize, store, and distribute plasma-activated liquids addresses some of the challenges associated with direct plasma application, particularly for widespread use in resource-limited settings [87]. Ongoing research is optimizing activation protocols and characterizing the stability and safety profiles of different plasma-activated liquids for specific clinical applications [88].

In dermatology, plasma-activated solutions are being investigated for treatment of skin infections and inflammatory conditions [86,87]. The ability to standardize, store, and distribute plasma-activated liquids addresses some of the challenges associated with direct plasma application, particularly for widespread use in resource-limited settings [87]. Ongoing research is optimizing activation protocols and characterizing the stability and safety profiles of different plasma-activated liquids for specific clinical applications [88].

### Biosafety, dosimetry, and standardization challenges

The clinical translation of CAP technology requires careful attention to biosafety considerations and the development of standardized dosimetry approaches. A well-documented feature of CAP is its selective toxicity towards microbial cells compared with mammalian cells. This selectivity arises from fundamental differences in cellular structure and physiology. Microbial cells have higher surface-to-volume ratios, membranes richer in oxidation-sensitive unsaturated lipids, and less sophisticated antioxidant defense systems compared with human cells. These differences create a therapeutic window where microbial inactivation can be achieved without significant damage to host tissues [8].

The selective susceptibility of micro-organisms vs mammalian cells to CAP arises from fundamental structural and physiological differences: microbial cells have a higher surface-to-volume ratio, membranes richer in oxidation-sensitive unsaturated lipids, and less sophisticated antioxidant defences, as demonstrated in comparative studies of CAP-treated mammalian and bacterial cells [89,90]. However, the absolute safety of CAP is not uniform – it is critically influenced by the choice of feed gas. Air-fed plasma sources can generate ozone (O_3_) and nitrogen oxides (NO_X_), which at high concentrations may irritate the respiratory tract if not properly exhausted. In contrast, noble gases such as helium or argon produce minimal ozone and fewer toxic by-products, but their extraction is energy-intensive. A 2025 pig model study confirmed that helium generates significantly less heat and fewer pathological changes than argon under identical settings, emphasizing that each device–gas combination requires independent safety validation [90]. Clinical-grade CAP systems are designed with effective gas capture or exhaust ventilation. To date, moderate treatment durations (2–5 min) are safe in animal models, and no mutagenic potential has been demonstrated for argon plasma jets under standard test conditions (Ames test). However, intense treatment can induce measurable DNA damage *in vitro*, highlighting the need for cautious selection of plasma intensity [89]. Standardization efforts have made progress: DIN SPEC 91315:2025-07 provides general requirements for characterizing medical plasma sources, and the standards for medical plasma sources (DINPlas) project aims to establish uniform test procedures [91]. Looking forwards, formal safety limits must be defined for each clinical indication, and future studies should systematically report gas composition, treatment parameters, exhaust handling, and any adverse events to enable proper risk assessment and device comparison. On the other hand, although individual clinical studies report only mild, transient adverse events, a quantitative synthesis of safety data (e.g. pooled incidence of adverse events) was not feasible due to heterogeneous reporting and short follow-up durations. Long-term prospective safety registries are urgently needed.

Clinical studies with medical-grade plasma devices have consistently demonstrated excellent safety profiles, with adverse events typically limited to transient, mild local reactions [92]. However, as with any medical intervention, appropriate dosing is essential. The primary challenge in CAP dosimetry is the complex nature of the ‘plasma dose,’ which encompasses multiple physical, chemical, and biological parameters [93]. Unlike conventional drugs with defined concentrations, the biological effects of CAP depend on factors, including power settings, treatment time, distance from source, gas composition, and environmental conditions [93]. Current research is focused on developing integrated dosimetry concepts that correlate these input parameters with biological outcomes, potentially using chemical dosimeters or biological indicators to standardize treatments across different devices and applications [94].

Standardization efforts are also addressing device classification, treatment protocols, and training requirements for clinical users [95]. Regulatory agencies in various regions have established pathways for medical plasma device approval, with several devices now having received clearance for specific indications [96]. These regulatory milestones represent important steps towards mainstream clinical adoption, but broader implementation will require continued collaboration between researchers, clinicians, industry partners, and regulatory bodies to establish evidence-based guidelines and best practices [97].

## Discussion

The evidence compiled in this review positions CAP as an innovative technology with significant potential in antimicrobial therapy. By leveraging a multi-target, physical–chemical–based mechanism of action – distinct from the single-target approach of conventional antibiotics – CAP achieves broad-spectrum efficacy while presenting a formidable barrier to resistance development. This is particularly critical in an era of stagnating antibiotic discovery [21]. Despite this transformative potential, Figure 2 illustrates that the path to widespread clinical adoption is contingent upon successfully addressing key hurdles in dosimetry standardization, long-term safety, and economic viability.

**Figure 2:**
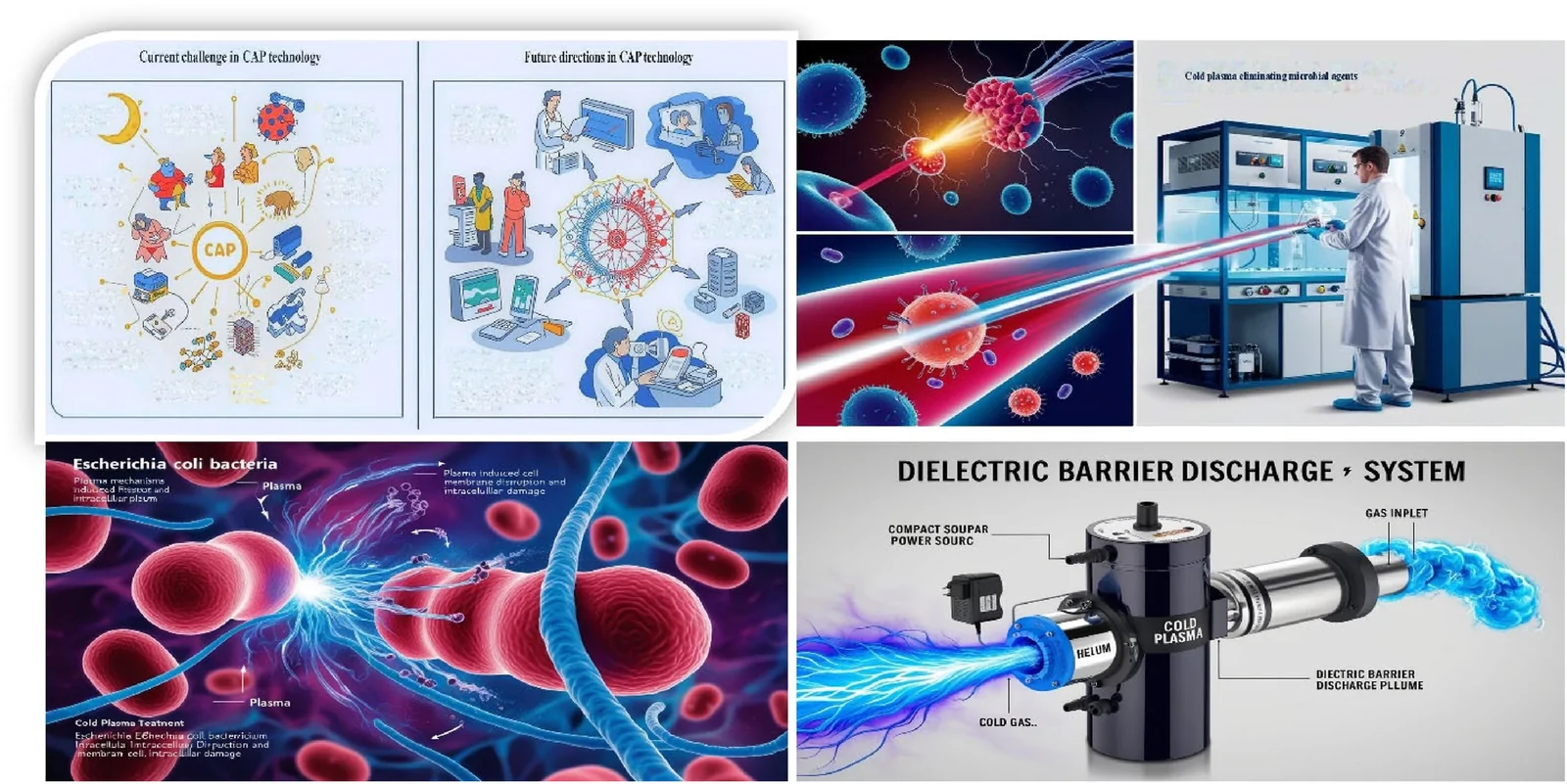
Key challenges and promising future directions for the clinical translation and advancement of cold atmospheric plasma (CAP) technology. Top left: future directions; top right: plasma jet treatment of a wound; bottom left: biofilm disruption; bottom right: schematic of dielectric barrier discharge (DBD). The figure outlines the primary hurdles currently impeding widespread adoption, including the need for standardized dosimetry, long-term safety data, and cost-effectiveness analyses, alongside strategic research priorities such as device miniaturization, exploration of combination therapies, and expansion into new clinical applications. All illustrations in this figure are original schematic designs created by the authors using Canva AI and Adobe Illustrator 2025; no third-party copyrighted material is included.

The comparative analysis reveals several distinctive advantages of CAP over conventional antimicrobial approaches. First, its efficacy against biofilms addresses a major limitation of systemic antibiotics, which is that it often fails to penetrate the extracellular polymeric substance matrix [28]. Second, the immediate microbial reduction achieved by CAP provides rapid control of infection burden, complementing the slower, growth-dependent action of many antibiotics [37]. Third, the ability to apply CAP topically enables localized treatment with minimal systemic exposure, reducing the risk of side-effects and drug interactions [98]. Fourth, the stimulation of tissue repair processes positions CAP as not merely an antimicrobial but a comprehensive wound therapy modality [99]. These advantages are not merely theoretical; they are increasingly supported by quantitative evidence from clinical trials.

The clinical translation of CAP technology demonstrates both significant progress and important limitations. Among these challenges, the lack of standardized dosimetry represents the most fundamental technical barrier. Without a universally accepted metric for the ‘plasma dose,’ comparing outcomes across studies and establishing definitive treatment protocols remain profoundly difficult [100]. While cost and reimbursement are significant hurdles for widespread adoption, they are secondary to the primary need for dosimetry standardization, which is a prerequisite for generating the robust, reproducible efficacy data required by payers and regulatory bodies [101].

The strongest evidence exists for chronic wound management, where multiple RCTs show consistent benefits [25]. This evidence has now been consolidated in a quantitative meta-analysis of 12 RCTs (477 participants), which reported significant reductions in wound area (MD = −1.36; 95% CI: −2.09 to −0.62; *P* = 0.0011) and bacterial load (MD = −0.36; 95% CI: −0.64 to −0.08; *P* = 0.0126), with a healing rate ratio of 2.90 (95% CI: 1.45–5.80; *P* = 0.0052) [10]. However, the heterogeneity in study designs, plasma devices, and treatment protocols complicates direct comparison and meta-analysis. This highlights the urgent need for standardized treatment protocols and consensus definitions of plasma dosage in clinical contexts.

When we synthesize evidence across wound healing, dental applications, antiviral activity, biofilm control, and safety/economics – rather than examining each in isolation – several previously unrecognized patterns emerge:

First, the therapeutic window of CAP is domain-dependent. The quantitative evidence for wound healing contrasts sharply with the predominantly preclinical evidence base for dental applications. A systematic review of 19 studies (11 in-vitro, 7 animal, and 1 human RCT) confirmed that CAP demonstrates consistent antimicrobial activity against oral pathogens, enhances bonding and remineralization, and shows no adverse histological effects; however, clinical evidence remains limited, and routine use cannot yet be recommended [11]. This disparity – robust quantitative evidence for wound healing vs limited clinical evidence for dentistry – suggests that CAP’s translational readiness varies substantially by indication, a nuance lost in single-domain reviews.

Second, plasma technology and carrier gas selection are not merely technical details – they fundamentally determine therapeutic outcomes. Subgroup analyses from the wound healing meta-analysis reveal that plasma jet technology yields a substantially larger effect size for healing rate (11.50; 95% CI: 2.91–45.49) than DBD (1.98; 95% CI: 1.39–2.80). Similarly, indirect atmospheric plasma and argon gas show significant bacterial load reduction (effect size:−0.72; 95% CI: −1.17 to −0.28; *P* < 0.01), whereas plasma jet and helium do not [36]. In dental applications, helium-based and air-based plasma jets demonstrate consistent antimicrobial effects, but the heterogeneity in devices and exposure durations precludes direct comparison [77]. This cross-domain analysis reveals that no single ‘CAP’ exists – rather, there is a family of plasma technologies with distinct efficacy profiles that must be matched to specific clinical indications.

Third, the evidence base is characterized by a ‘preclinical–clinical gap’ that varies by domain. In wound healing, the evidence has progressed to multiple RCTs and meta-analyses with quantitative effect estimates. In dentistry, the evidence remains heavily weighted towards in-vitro and animal studies, with only one short-term RCT available [10,11]. This gap is not merely academic – it has direct implications for clinical translation, reimbursement, and guideline development. The wound healing data are sufficiently robust to support regulatory approvals and clinical adoption, whereas dental applications require substantial further clinical validation before routine use can be recommended.

Fourth, safety data are consistent across domains but limited in duration. The 12-month mouse study by Evert *et al.* (2021) demonstrated no carcinogenic or dysplastic changes following repeated oral mucosa exposure [11]. In wound healing, clinical trials report only mild, transient adverse events [10]. However, as noted in the present review, a quantitative synthesis of safety data (e.g. pooled incidence of adverse events) was not feasible due to heterogeneous reporting and short follow-up durations. Long-term prospective safety registries are urgently needed across all domains.

Fifth, the economic and regulatory landscape is fragmented. While CAP devices have received CE marking in Europe for wound healing (kINPen® MED, neoplasmed GmbH, Greifswald, Germany, class IIa since 2013) and limited US Food and Drug Administration clearance in the USA for soft tissue ablation, no globally harmonized standard exists. The heterogeneity in plasma devices, gases, and protocols – documented in both wound healing [10] and dental [11] reviews – underscores the urgent need for standardized treatment protocols and consensus definitions of plasma dosage.

The application of CAP in healthcare has garnered significant attention, particularly concerning its multi-faceted use in combating HAIs. Previous scholarly examinations have meticulously explored various dimensions of CAP’s efficacy, specifically its role in biofilm control and in reducing bacterial load [6,7,9]. However, none have integrated quantitative wound healing data [10], dental preclinical evidence [11], antiviral activity, biosafety, and economic considerations within a single framework. The present review fills this gap by demonstrating that the translational readiness of CAP varies substantially by indication, that plasma technology selection is a critical determinant of efficacy, and that standardization remains the single most important barrier to widespread adoption. This integrated perspective offers a more complete, critical, and practically useful synthesis for researchers, clinicians, policymakers, and health economists alike.

Our findings align with and extend several previously published reviews. O'Connor *et al.* [6] emphasized CAP’s potential for hospital decontamination but noted the lack of standardized protocols – a limitation we confirm and expand upon by quantifying the heterogeneity in plasma sources and treatment parameters across 64 studies. Boyce [7] discussed emerging disinfection technologies including CAP, but without a systematic synthesis of clinical data; our review addresses this gap by integrating quantitative wound healing outcomes (MD = −1.36; risk ratio = 2.90) with mechanistic insights. Li *et al.* [8] summarized bacterial load reduction in wounds; our analysis confirms this effect and further demonstrates that bacterial load reduction is significantly modulated by plasma technology and carrier gas selection (e.g. indirect atmospheric plasma and argon show significant effects, whereas plasma jet and helium do not). Mai-Prochnow *et al.* [9] reviewed biofilm interactions from a mechanistic perspective; we extend this by showing that CAP achieves biofilm eradication where conventional antiseptics fail and by linking these effects to clinical outcomes. Importantly, while these earlier reviews provided valuable foundations, they were published between 2014 and 2016 and did not integrate recent mechanistic advances (e.g. electric fields, apoptosis-like responses), updated clinical trials, biosafety, dosimetry, economic, and environmental considerations. Our review fills this gap by providing a comprehensive, integrated synthesis that connects mechanisms, efficacy across all pathogen classes, clinical translation, safety, and economic factors within a single framework.

Beyond the direct capital outlay, the broader regulatory, economic, and environmental implications of CAP technology warrant careful consideration. Regarding the regulatory landscape, medical plasma devices have received CE marking in Europe for wound healing applications, with kINPen® MED representing one of the best-known clinically translated examples [75,81,91]. However, while preliminary technical standards exist, such as DIN SPEC 91315:2025-07 in Germany, no globally harmonized clinical and regulatory standard for CAP has yet been established, and each new indication still requires separate validation [38,91,102].

The choice of feed gas also has direct safety and environmental implications. Argon is chemically inert and widely used in plasma jets, whereas air-fed systems may reduce dependence on external gas supply but can present occupational safety concerns due to the local generation of ozone (O_3_) and nitrogen oxides (NO_x_), which require adequate scavenging or ventilation systems [89,102,103]. More broadly, environmental life-cycle considerations of CAP systems remain underexplored in the current literature [3,17].

Economically, in many healthcare systems, reimbursement for CAP therapy remains restricted to specific indications such as chronic wounds and may still depend on local clinical adoption pathways or evaluation frameworks [81,92,99]. By contrast, conventional wound dressings and antiseptics are inexpensive and widely available, creating a high bar for new technologies to demonstrate cost-effectiveness. For CAP to transition from specialized centres to routine clinical use, formal health economic analyses are needed to determine whether faster wound closure, reduced infection-related hospitalizations, and lower antibiotic consumption can offset the upfront investment [81,99]. Encouragingly, recent modelling studies suggest that device-assisted plasma treatment may be cost-saving in selected chronic wound care settings, particularly when improved healing trajectories and fewer secondary complications are considered, although such estimates remain highly context-dependent and require validation across diverse clinical environments [81,92,99].

Moreover, consumables such as gas cartridges and disposable nozzles can increase per-treatment expenses [38,81], while maintenance, calibration, and component replacement may further affect long-term operating costs [76]. This combination of upfront costs and recurring expenses remains a barrier, particularly in resource-limited settings. Additionally, maintaining plasma uniformity and reactive chemistry across large or irregular treatment areas poses a significant technical scalability challenge that can directly influence treatment time, reproducibility, and clinical throughput [38,76,93,95]. Addressing these regulatory, safety, economic, and logistical barriers will be critical for the equitable scaling of CAP technology. Conducting a formal cost-effectiveness analysis was beyond the scope of this qualitative review; however, the available modelling literature suggests that CAP could become cost-saving in specific indications if upfront device costs and consumables are reduced [81,92,99].

When positioned within the broader landscape of non-antibiotic antimicrobial strategies, CAP’s unique value proposition becomes clear. Unlike photodynamic therapy (PDT), CAP does not require exogenous photosensitizers, simplifying treatment logistics and eliminating potential photosensitivity in patients [104]. Compared with antimicrobial peptides (AMPs), which can have high production costs and susceptibility to proteolytic degradation, CAP offers immediate, on-demand activity with no risk of enzymatic inactivation. While ultrasound-based methods can disrupt biofilms physically, they often lack the potent, complementary chemical action provided by CAP’s RONS [105,106]. However, each technology has its niche; PDT offers exceptional spatial precision, AMPs can be systemically administered, and ultrasound provides deep tissue penetration. This suggests that the future may lie not in competition, but in combination, where CAP could be used to precondition biofilms or enhance the penetration of subsequently applied agents like AMPs or antibiotics, creating powerful synergistic treatment regimens.

The integration of CAP with established antimicrobial stewardship programmes represents a promising strategy. Rather than positioning CAP broadly as a first-line therapy, it may be more appropriate to consider CAP as an adjunctive modality or, in selected superficial and localized infections, a potential alternative to systemic antibiotics, thereby preserving systemic antibiotics for more serious indications. Additionally, CAP's ability to treat infections caused by highly drug-resistant organisms, including pathogens with limited therapeutic options, provides a valuable option when conventional therapies have failed. This approach aligns with the broader goal of preserving the efficacy of existing antibiotics through reduced usage.

Despite the strengths of this integrated approach, several limitations must be acknowledged. First, a quantitative meta-analysis was not performed because substantial heterogeneity in plasma sources, exposure parameters, biological models, and outcome definitions would have limited the interpretability of pooled estimates; instead, we provided a qualitative synthesis across 64 studies, informed by the quantitative findings of the recent meta-analysis by Yuan *et al.* [10]. Second, the majority of included studies (*N* = 47) were in-vitro mechanistic investigations, limiting direct clinical extrapolation and reducing confidence in the immediate generalizability of findings to patient care settings. Third, heterogeneity across plasma systems, including power, gas composition, exposure distance, humidity, treatment duration, and biological endpoints, complicates direct comparison. The decision to limit the search to publications from 2018 onwards was deliberate and based on two considerations. First, the foundational work on CAP’s antimicrobial mechanisms and early clinical applications was comprehensively summarized in several high-quality systematic and narrative reviews published between 2014 and 2016, making duplication unnecessary. Second, the period from 2018 to the present has witnessed a marked acceleration in the translation of CAP from preclinical models to clinical practice, with the emergence of commercial medical plasma devices, RCTs, and standardization initiatives – all of which form the core focus of this review. Fourth, publication bias, suggested by funnel plot asymmetry in the wound healing meta-analysis [10], may have inflated effect estimates. Fifth, the absence of PROSPERO registration is a limitation; however, to mitigate this, we ensured transparency by explicitly reporting the search strategy, eligibility criteria, study selection process, and data extraction approach. Sixth, we did not produce a formal risk-of-bias table using Cochrane RoB 2 or SYRCLE’s tools because the vast majority of included studies were in-vitro mechanistic investigations for which such tools are not designed. Instead, we emphasize that reproducibility-related reporting elements, such as device parameters, exposure distance, treatment duration, temperature control, and outcome definitions, are particularly important for assessing the interpretability of in-vitro CAP studies.

From a technological perspective, several research priorities emerge. The development of real-time monitoring systems to quantify clinically relevant CAP exposure using measurable surrogate parameters would address a critical limitation in current practice. These may include surface power density, gas composition, temperature, and RONS-related chemical markers, which together may enable more reproducible dose–response characterization. The optimization of plasma sources for specific applications, such as deep tissue penetration or large surface area coverage, requires continued engineering innovation. Furthermore, the exploration of combination therapies with conventional antimicrobials, immunomodulators, or other physical modalities represents a promising research direction. However, such combinations will require a careful evaluation of potential drug oxidation, sequencing effects, and tissue toxicity thresholds.

The future development of CAP technology will likely proceed along several parallel tracks: (1) Technologically, the trajectory points towards the development of flexible, wearable plasma patches for at-home management of chronic wounds, and the integration of real-time biosensors for closed-loop dose control; (2) Clinically, promising frontiers include the development of catheter-based plasma systems for treating endovascular infections, inhaled plasma-activated aerosols for respiratory tract decontamination, and its use in veterinary medicine to combat infections in livestock, thereby reducing agricultural antibiotic use; (3) From a commercial perspective, the development of disposable or single-use plasma devices could address infection control concerns while expanding accessibility. Because these applications differ substantially in technical readiness and safety requirements, their translation will likely occur at different paces, with superficial wound applications representing a nearer-term opportunity than endovascular or inhalational approaches.

The strategic implementation of CAP technology should consider both technological and socio-economic factors. In high-resource settings, CAP could be integrated into specialized wound care centres and operating rooms for procedural sterilization. In low-resource settings, simpler, more affordable devices could address basic infection control needs. Educational initiatives targeting both healthcare providers and patients will be essential for successful adoption. Furthermore, health economic studies demonstrating cost-effectiveness will be crucial for securing reimbursement and institutional support. Such studies should ideally evaluate outcomes such as reduced systemic antibiotic consumption, faster wound closure, lower recurrence rates, fewer hospital re-admissions, and overall cost per successfully treated case.

This systematic review provides comprehensive evidence that CAP technology represents a transformative approach to antimicrobial therapy with significant potential to address the global challenge of AMR. The mechanistic insights, efficacy data, and clinical evidence synthesized in this review support several key conclusions.

First, CAP’s multi-target mechanism of action, primarily mediated by RONS, provides broad-spectrum antimicrobial activity against diverse pathogens, including multi-drug-resistant bacteria, viruses, fungi, and biofilms. The integration of chemical, electrical, and biological effects creates a comprehensive antimicrobial approach that is difficult for micro-organisms to counteract through conventional resistance mechanisms.

Second, the clinical applications, particularly in wound management and dentistry, demonstrate tangible benefits and an excellent safety profile. The dual action of microbial reduction and tissue regeneration stimulation positions CAP as a comprehensive therapeutic modality rather than merely a disinfectant. The ongoing regulatory approvals and technological advancements indicate steady progress towards mainstream clinical adoption.

Third, while challenges in standardization and dosimetry remain, the research community has made significant strides towards overcoming these limitations. The development of plasma-activated liquids, improved device designs, and better understanding of biological mechanisms are gradually overcoming the barriers to widespread implementation.

In summary, the present review offers several novel contributions: (i) it synthesizes mechanistic advances over the past six years, including the role of electric fields and apoptosis-like responses; (ii) it provides a systematic, comparative evaluation of efficacy across all major pathogen classes (bacteria, viruses, fungi, spores, biofilms) using standardized log-reduction metrics; (iii) it updates clinical translation with data from recent RCTs not covered in earlier reviews; (iv) it critically analyses biosafety, dosimetry, and the emerging field of plasma-activated liquids; and (v) it articulates five previously unrecognized cross-domain patterns that demonstrate original insight – namely, that the therapeutic window of CAP is domain-dependent, that plasma technology and carrier gas selection fundamentally determine outcomes, that a preclinical–clinical gap varies by domain, that safety data are consistent but limited in duration, and that the economic and regulatory landscape is fragmented. To our knowledge, no previous systematic review has integrated these dimensions within a single framework focused on AMR.

Looking forward, the successful integration of CAP into clinical practice will require continued interdisciplinary collaboration, technological innovation, and clinical validation. As research continues to refine applications and establish evidence-based guidelines, CAP is poised to become an increasingly important component of the antimicrobial arsenal, contributing to global efforts to preserve the efficacy of antimicrobial therapies for future generations.

## CRediT authorship contribution statement

**Amir Namvar Vansofla:** Writing – original draft, Methodology, Investigation, Formal analysis, Data curation. **Mohammad Ebrahim Minaei:** Visualization, Validation, Data curation, Conceptualization. **Neda Yousefi Nojookambari:** Visualization, Investigation, Data curation. **Sajjad Yazdansetad:** Writing – review & editing, Validation, Supervision, Project administration.

## Ethical approval

Not required.

## Funding sources

This research did not receive any specific grant from funding agencies in the public, commercial, or not-for-profit sectors.

## Conflict of interest statement

The authors declare no conflict of interest.
